# Mucosal delivery of anti-inflammatory IL-1Ra by sporulating recombinant bacteria

**DOI:** 10.1186/1472-6750-4-27

**Published:** 2004-10-30

**Authors:** Stefano Porzio, Paola Bossù, Paolo Ruggiero, Diana Boraschi, Aldo Tagliabue

**Affiliations:** 1 Inpharzam Ricerche SA, Zambon Group, Via ai Söi, CH-6807 Taverne, Switzerland; 2 Lab. Experimental Neuro-Psychobiology, Clinical and Behavioral Neurology, IRCCS Fondazione S. Lucia, Via Ardeatina 306, I-00179 Roma, Italy; 3 IRIS Research Center, Chiron Srl, Via Fiorentina 1, I-53100 Siena, Italy; 4 Institute of Biomedical Technologies, CNR, Via G. Moruzzi 1, I-56124 Pisa, Italy; 5 ALTA S.r.l., Via Nino Bixio 15, I-53100 Siena, Italy; 6 on leave to the International Vaccine Institute, SNU Research Park, San 4–8 Bongcheon-7 dong, Kwanak-gu, Seoul 151-818, Korea

## Abstract

**Background:**

Mucosal delivery of therapeutic protein drugs or vaccines is actively investigated, in order to improve bioavailability and avoid side effects associated with systemic administration. Orally administered bacteria, engineered to produce anti-inflammatory cytokines (IL-10, IL-1Ra), have shown localised ameliorating effects in inflammatory gastro-intestinal conditions. However, the possible systemic effects of mucosally delivered recombinant bacteria have not been investigated.

**Results:**

*B. subtilis *was engineered to produce the mature human IL-1 receptor antagonist (IL-1Ra). When recombinant *B. subtilis *was instilled in the distal colon of rats or rabbits, human IL-1Ra was found both in the intestinal lavage and in the serum of treated animals. The IL-1Ra protein in serum was intact and biologically active. IL-1-induced fever, neutrophilia, hypoglycemia and hypoferremia were inhibited in a dose-dependent fashion by intra-colon administration of IL-1Ra-producing *B. subtilis*. In the mouse, intra-peritoneal treatment with recombinant *B. subtilis *could inhibit endotoxin-induced shock and death. Instillation in the rabbit colon of another recombinant *B. subtilis *strain, which releases bioactive human recombinant IL-1β upon autolysis, could induce fever and eventually death, similarly to parenteral administration of high doses of IL-1β.

**Conclusions:**

A novel system of controlled release of pharmacologically active proteins is described, which exploits bacterial autolysis in a non-permissive environment. Mucosal administration of recombinant *B. subtilis *causes the release of cytoplasmic recombinant proteins, which can then be found in serum and exert their biological activity *in vivo *systemically.

## Background

The use of recombinant proteins as drugs has deeply modified the therapeutic approach to many severe diseases. However, a variety of practical problems limits the use of biotechnological protein drugs. Stability of the active proteins, need for parenteral administration, and high costs of the final purified materials are among the most significant drawbacks. A way of circumventing these issues is represented by the direct administration of recombinant bacteria, acting simultaneously as cell factory and delivery system for pharmacologically active proteins. This approach has been already extensively experimented for the mucosal delivery of vaccine antigens [1,2]. In recent years, the local delivery of therapeutic antibodies [3,4], adjuvant cytokines [5,6], and anti-inflammatory cytokines [7-9] has been successfully attempted with food-grade bacteria (*e.g.*, *Lactococcus lactis*, *Streptococcus gordonii*), although limited to the therapy of localised pathologies (*e.g.*, inflammatory bowel diseases, IBD, in the gastro-intestinal tract).

Among anti-inflammatory strategies, both at systemic and local level, the use of the IL-1 receptor antagonist (IL-1Ra) has received vast attention. IL-1 is a family of cytokines highly active in the modulation of immune amplification and inflammation. The IL-1 family includes two agonist proteins, IL-1α and IL-1β, and one antagonist protein, IL-1Ra. IL-1β is a very potent immunostimulatory and inflammatory cytokine, responsible for initiating and amplifying the host response to invasion. If not properly controlled, IL-1 can cause fever, acute inflammation, tissue destruction, organ failure, and eventually shock and death (reviewed in [10]). IL-1Ra inhibits IL-1 by acting as a competitive receptor antagonist with no detectable agonist activity, thus representing a natural powerful mechanism to control IL-1-dependent responses and avoid pathological derangement (reviewed in [11,12]). In experimental animal models, IL-1Ra has demonstrated excellent therapeutic effects against acute and chronic inflammatory pathologies, being also effective at high doses in prolonging survival in endotoxic shock [11-17]. In human trials, IL-1Ra has been administered to patients with septic shock, rheumatoid arthritis, graft-*versus*-host disease, and multiple sclerosis (reviewed in [11,12,16]). While only a modest benefit was achieved in patients with septic shock [11,12,16,18], IL-1Ra had a clear beneficial effect in reducing joint destruction in rheumatoid arthritis [11,12,19-21]. From the clinical experience with purified recombinant IL-1Ra it became clear that most of the problems of variability of efficacy were due to difficulties in adequate timing and dosage of the drug [12]. To overcome these problems, gene therapy with adenoviral vectors carrying the IL-1Ra gene has been attempted in experimental animals, yielding promising results in models of type 1 diabetes and ischemic brain damage [22,23]. The clinical application of the gene therapy approach may however meet with difficulties for safety reasons, besides the problems of controlling drug release, concentration, and localisation.

Based on previous experience of using recombinant bacteria as *in vivo *cell factory, here we describe a novel system of local delivery of IL-1Ra, able to achieve systemic effects. The system exploits the ability of certain bacteria (such as *Bacillus subtilis*) to undergo autolysis in non-permissive conditions (as it occurs in the mammalian intestine) thereby releasing the cytoplasmic proteins. Intra-colon instillation of *B. subtilis *expressing recombinant human mature IL-1Ra induces significant serum levels of the recombinant protein in rats and rabbits, and prevents the inflammatory effects of systemic IL-1. Intra-peritoneal administration of recombinant *B. subtilis *in the mouse could inhibit LPS-induced shock and death. Further experimental evidence with a *B. subtilis *strain producing human IL-1β demonstrates that this delivery system can be generalised to other recombinant proteins.

## Results

**Figure 1:**
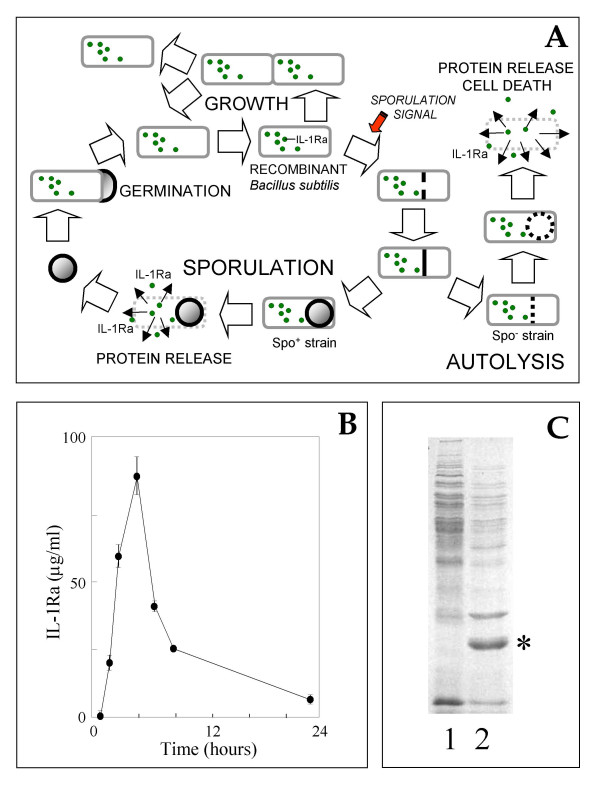
Release of IL-1Ra from recombinant *B. subtilis*. A. Schematic representation of the life cycle of *B. subtilis *showing the mechanism of recombinant IL-1Ra release upon sporulation (autolysis with spore formation, as in Spo^+ ^strain), and destructive autolysis (with cell lysis without spore formation, as in Spo^- ^strain). B. Presence of recombinant IL-1Ra in sporulation supernatant of pSM539 Spo^+ ^strain, assessed by ELISA (mean ± SEM). IL-1Ra amount in sporulation supernatants was also quantitatively evaluated by laser scanning densitometry. The amount of IL-1Ra released *in vitro *by 0.5–0.7 × 10^9 ^bacteria (0.25 g wet weight) 3 h after the sporulation signal was 1.152 mg in 9.6 mg total released proteins (*i.e.*, 12% of the total protein in the sporulation supernatant). Overlapping results were obtained with Spo^- ^pSM539 cells (data not shown). C. SDS-PAGE analysis of wild-type *B. subtilis *(lane 1) and of recombinant *B. subtilis *pSM539 Spo^- ^strain expressing IL-1Ra (lane 2). Asterisk indicates the migration position of human mature IL-1Ra. The protein identity was confirmed in Western blotting. Identical results were obtained with the Spo^+ ^strain (data not shown).

The ability of *B. subtilis *to generate spores by autolysis of the cell wall, thereby releasing cytoplasmic proteins, was exploited as system for delivery of proteins *in vivo*. Following a sporulation signal (*e.g.*, nutrient depletion) bacteria undergo an autolytic process with release of most of their cellular components and formation of a highly resistant spore containing DNA and few essential proteins (Figure 1A). As already shown in a previous study [24], *in vitro *sporulation of *B. subtilis *engineered for endocellular expression of human IL-1Ra (strain pSM539) caused the release of large amounts of intact and active recombinant protein within a few hours after the sporulation signal (Figure 1B). The *in vitro *release of intracellular recombinant IL-1Ra was equally evident in pSM539 Spo^+ ^(normally sporulating) and Spo^- ^bacteria, *i.e. *genetically modified cells which, in response to the sporulation signal, start the autolysis process but are unable to form a complete spore thus undergoing complete cell destruction (Figure 1C). Both Spo^+ ^and Spo^- ^strains of pSM539 were used in subsequent *in vivo *experiments with identical results.

The IL-1Ra recovered after *B. subtilis *autolysis *in vitro *retained full biological activity, with a specific activity of 1.1 × 10^6 ^inhibitory units (IU)/mg *vs. *0.9 × 10^6 ^IU/mg of reference standard IL-1Ra [24]. To assess whether the recombinant protein could also be released *in vivo *by engineered *B. subtilis*, the bacterial strain engineered with IL-1Ra was administered intra-peritoneally in the mouse, in the small and large intestine of rats, and in the rabbit distal colon. The presence of human IL-1Ra was assessed at different times after administration, both locally and in the serum of treated animals, by Western blotting, ELISA, and BIAcore analysis (Table 1). Intact human IL-1Ra was found locally at high levels 3 hours after administration of recombinant bacteria and persisted for several hours. In the serum, intact human IL-1Ra could be found at measurable levels when *B. subtilis *was administered intra-peritoneally (2/2 mice) or in the colon (5/9 rats, 27/31 rabbits), but not when bacteria were administered in the small intestine (0/5 rats).

**Figure 2:**
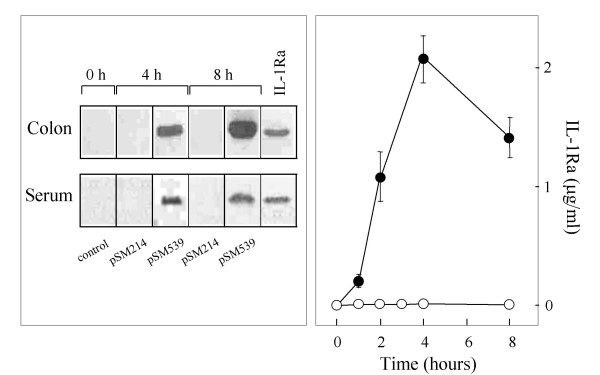
Presence of intact IL-1Ra in serum after intra-colonic administration of IL-1Ra-producing *B. subtilis*. Left: Human IL-1Ra in colon washings and in serum of rats receiving a single intra-colonic instillation of 3 × 10^8 ^live cells of *B. subtilis *pSM214 (β-lactamase control) or pSM539 (producing IL-1Ra). The presence of IL-1Ra was assessed by Western blotting on samples of colon washings (upper panel) and serum (lower panel) taken at different time points. Samples at time 0 were from animals receiving pSM539 immediately before sampling. Results at time 0 from rats treated with pSM214 and untreated rats were also negative (data not shown). An aliquot of standard human recombinant IL-1Ra (7–14 ng) was included as reference in each gel. Right: Presence of human IL-1Ra in serum of rabbits receiving an intra-colonic instillation of 2 × 10^9 ^live cells of *B. subtilis *pSM539 (producing IL-1Ra; ●) or pSM214 (β-lactamase control;○). IL-1Ra concentration was assessed by ELISA. Data are the mean ± SEM of values from 3 rabbits/group in one representative experiment. Overlapping results were obtained with sera of rats after intra-colonic administration of recombinant *B. subtilis*, and by BIAcore analysis of human IL-1Ra presence in rat and rabbit serum samples (data not shown). Statistical significance: pSM539 *vs*. pSM214 p < 0.01 at every time.

The delivery of IL-1Ra at the intestinal mucosal level was examined. Live cells of the IL-1Ra-producing pSM539 strain were instilled in the rat distal colon, a non-permissive environment that does not allow *B. subtilis *vegetative life [26]. As a control, animals received equal numbers of the pSM214 strain, *i.e. B. subtilis *cells transformed with the β-lactamase-expressing control plasmid pSM214. Data in Figure 2 show that the presence of intact IL-1Ra can be measured both locally (in the intestinal washings) and in serum for several hours after intra-colonic inoculum of the IL-1Ra-producing *B. subtilis *strain pSM539, whereas serum of animals receiving *B. subtilis *pSM214 remained negative. As a control, pSM539 bacteria delivered in the small intestine released detectable amounts of IL-1Ra locally, but no IL-1Ra could be found at the serum level (data not shown; Table 1). The serum pharmacokinetic parameters of IL-1Ra released by recombinant *B. subtilis*, as compared to the purified protein administered intra-colonically, show a few differences (Table 2). The C_max _was higher and the T_max _quicker for the purified protein, as compared to IL-1Ra released from intra-colonically administered *B. subtilis*. On the other hand, the AUC/dose was almost identical. Administration of control bacteria pSM214 intra-colonically together with the purified protein did not significantly change the pharmacokinetics parameters of IL-1Ra, except for a slight decrease of the total dose absorbed, indicating that the physical presence of bacteria has little effect on IL-1Ra absorption. It is concluded that engineered *B. subtilis *delivered intra-colonically releases, conceivably by autolysis, the cytoplasmic recombinant protein, which is subsequently absorbed and can be detected intact at measurable levels in the bloodstream.

**Figure 3:**
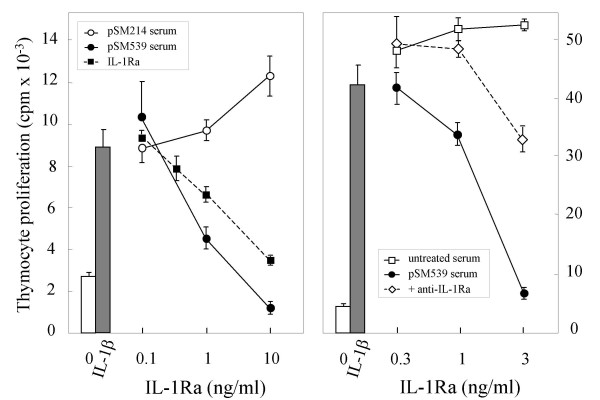
Serum IL-1Ra from intra-colonic *B. subtilis *inhibits IL-1-induced thymocyte proliferation *in vitro*. Co-stimulation of murine thymocyte proliferation by IL-1β was assessed in cultures of C3H/HeJ thymocytes stimulated with a suboptimal concentration of PHA (1.5 μg/ml) in the absence (zero control; empty column) or in the presence of IL-1β (solid column). Left panel: Serum samples were taken from rabbits administered intra-colonically 3 h earlier with *B. subtilis *pSM539 (producing IL-1Ra; ●) or pSM214 (β-lactamase control; ○), and assayed by ELISA for the presence of IL-1Ra (1.99 μg/ml in the pSM539 serum; 0.00 μg/ml in the pSM214 serum). Inhibition of IL-1β activity (30 pg/ml) was assessed as decrease of thymocyte proliferation in the presence of different dilutions of the pSM539 serum (1:200, 1:2,000, 1:20,000; corresponding to 10, 1, and 0.1 ng/ml IL-1Ra) and compared to the same concentrations of human recombinant IL-1Ra (■). Right panel: Thymocyte proliferation to IL-1β (300 pg/ml) was evaluated in the presence of rabbit serum taken 6 h after intra-colon administration of pSM539 (●) or serum from the same rabbit taken before treatment (□). Serum from pSM539-treated rabbit contained 1.35 μg IL-1Ra/ml (tested by ELISA). Serum was added at dilutions (1:450, 1:1,350, 1:4,050) containing 3, 1, and 0.3 ng/ml IL-1Ra, either alone (●) or in the presence of a polyclonal antibody against human IL-1Ra (diluted 1:300; ◇). Untreated rabbit serum (negative for IL-1Ra in ELISA) was used as control at the same dilutions as pSM539 serum. Results are presented as mean ± SEM of triplicate cultures within single representative experiments. Statistical significance: p < 0.01 pSM539 serum (at 1, 3, 10 ng IL-1Ra/ml) *vs*. IL-1β alone and corresponding serum controls (pSM214, untreated, + anti-IL-1Ra).

To verify that IL-1Ra found in serum after *B. subtilis *administration at the mucosal level is functional, its ability to inhibit IL-1 was evaluated both *in vitro *and *in vivo*. *In vitro*, activity of standard IL-1β was assessed with the classical co-stimulation assay on thymocytes of LPS-unresponsive C3H/HeJ mice. Inhibition by IL-1Ra was evaluated as capacity to decrease IL-1-induced thymocyte proliferation. The presence of biologically active IL-1Ra was measured as inhibition of IL-1β-induced thymocyte proliferation by the IL-1Ra-containing serum of rabbits administered pSM539 intra-colonically. The presence and amount of IL-1Ra was measured by ELISA in the serum of pSM539-treated rabbits and of control pSM214-treated or untreated animals. As shown in Figure 3, IL-1Ra-containing serum from pSM539-treated rabbit (used at dilutions containing from 0.1 to 10 ng/ml IL-1Ra) was as effective in inhibiting IL-1β activity as the same concentrations of standard purified recombinant IL-1Ra (Figure 3, left), whereas the same dilutions of serum from pSM214-treated or from untreated animals (devoid of IL-1Ra) did not possess any IL-1-inhibiting activity (Figure 3, left and right panels). To confirm that the IL-1β-inhibiting activity observed in sera of pSM539 treated animals is indeed due to IL-1Ra, data in the Figure 3 (right) show that the inhibitory capacity of pSM539 serum is significantly decreased or abolished by an antiserum against human IL-1Ra. Thus, it can be concluded that the IL-1Ra present in serum after intra-colonic administration of pSM539 is biologically active.

**Figure 4:**
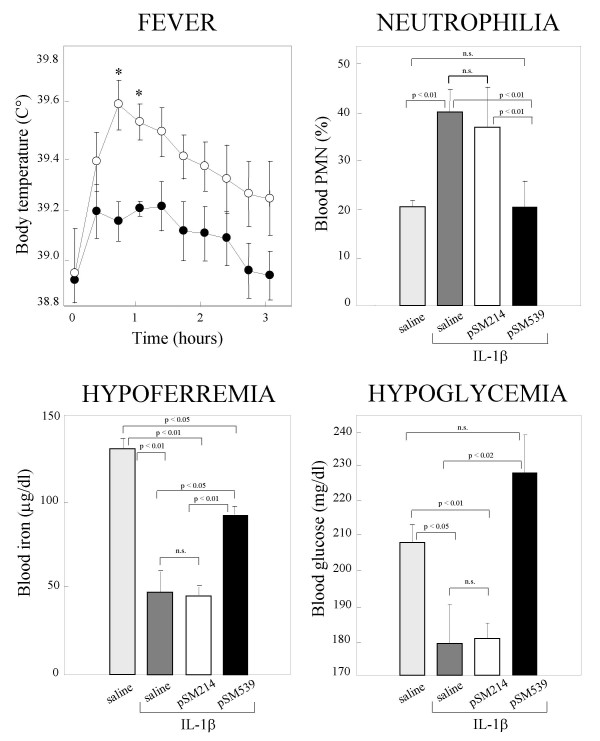
Inhibition of IL-1 systemic effects *in vivo *by intra-colonic administration of IL-1Ra-producing *B. subtilis*. Upper left: Increase in body temperature in rabbits treated with human recombinant IL-1β (75 ng/kg i.v.) 1 h after a intra-colonic instillation of 2 × 10^9 ^live cells of *B. subtilis *pSM539 (producing IL-1Ra; ●) or pSM214 (β-lactamase control; ○). Data are the mean ± SEM of values from 3 rabbits/group. Difference was statistically significant with p < 0.02 at 40 and 60 min. Upper right: Granulocytosis induced by human recombinant IL-1β in rats. Animals were administered intra-colonically with 1 ml/kg saline (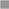) or with the same volume of saline containing 1 × 10^9 ^live cells/kg of pSM214 (□) or pSM539 (■). After 2 h animals received 100 ng/kg human recombinant IL-1β i.p. A control group received saline i.p. (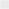) instead of IL-1β. All animals were bled 2 h later, and the number of circulating PMN was evaluated cytofluorimetrically. PMN numbers are expressed as percent blood leukocytes and reported as the mean ± SEM of values in 3 rats/group. Lower left: Hypoferremia induced by human recombinant IL-1β in rabbits. Animals received two intra-colonic instillations of 1 ml/kg saline alone (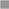) or containing 2 × 10^9 ^live cells of *B. subtilis *pSM539 (producing IL-1Ra; ■) or pSM214 (β-lactamase control; □), 3 h before and 10 min after administration of IL-1β (100 ng/kg i.p.). Control rabbits administered intra-colonically with saline received an i.p. inoculum of saline instead of IL-1β (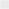). Serum iron levels were measured 8 h later. Data are the mean ± SEM of values of 3–25 rabbits, tested in five separate experiments and representative of results obtained at different times after IL-1β inoculum (4–24 h). Lower right: Hypoglycemia induced by human recombinant IL-1β in rats. Animals were administered intra-colonically with 1 ml/kg saline (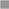) or with the same volume of saline containing 1 × 10^9 ^live cells/kg of pSM214 (□) or pSM539 (■). After 2 h animals received 100 ng/kg human recombinant IL-1β i.p. A control group received saline i.p. (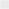) instead of IL-1β. All animals were bled 2 h later, and serum samples were assayed for glucose concentration. Data are reported as the mean ± SEM of values in 3 rats/group.

The *in vivo *efficacy of IL-1Ra released by intra-colonic pSM539 was evaluated in antagonising the effects of parenterally administered IL-1 [27]. As shown in Figure 4 (upper left), the increase in body temperature induced in rabbits by i.v. administration of 75 ng/kg human IL-1β was significantly reduced by preventive intra-colonic treatment with 2 × 10^9 ^cells of *B. subtilis *pSM539. The reduction of IL-1β-induced fever was more pronounced with lower doses of IL-1β (90% reduction of peak fever induced by 50 ng/kg IL-1β), but it was still highly significant when fever was induced by 100 ng/kg IL-1β (>60% reduction of peak fever) (data not shown). To confirm these data, the effect of intra-colonic treatment with IL-1Ra-producing pSM539 was evaluated on other inflammation-related parameters induced by IL-1β, *i.e.*, granulocytosis and decrease of blood glucose and iron concentrations. As shown in Figure 4 (upper right), the increase in circulating PMN induced in rats by IL-1β i.p. was abrogated by previous intra-colonic administration of IL-1Ra-producing pSM539 but not by the control strain pSM214. Likewise, the IL-1β-induced decrease in the blood levels of iron (Figure 4, lower left) and glucose (Figure 4, lower right) was still evident in animals administered control pSM214 bacteria but was significantly reduced by intra-colonic instillation of IL-1Ra-producing pSM539 bacteria. It is inferred that human recombinant IL-1Ra delivered *in vivo *by intra-colonic administration of engineered *B. subtilis *is biologically active and able to counteract the systemic inflammatory effects of IL-1.

**Figure 5:**
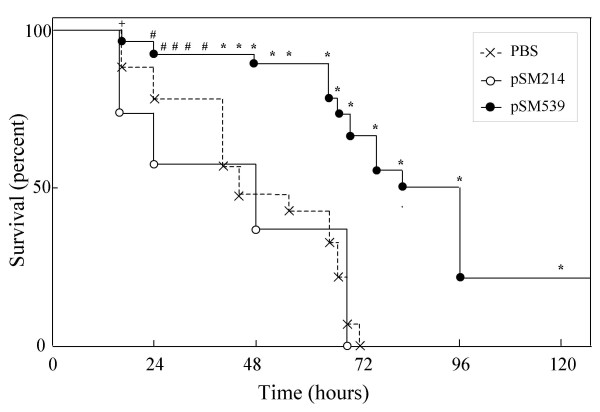
IL-1Ra-producing *B. subtilis *protects mice from endotoxic shock. LPS-sensitive C3H/HeOuJ mice (29–52 mice/group tested in four separate experiments) received 3 × 10^6 ^pSM539 bacteria (engineered with human mature IL-1Ra; ●) i.p. 24 h before administration of LPS (15–20 mg/kg). Mice were observed for 96 h and deaths recorded. Control mice received either the *B. subtilis *strain pSM214 (β-lactamase control;) or PBS (×) 24 h before LPS administration. Statistical analysis of survival curves (χ^2^) indicated a significant increase in survival of mice receiving pSM539 as compared to controls receiving PBS or receiving pSM214. At 16 h difference was statistically significant at p < 0.05 *vs*. pSM214 but not significant *vs*. PBS (+). From 24 to 40 h, difference was statistically significant only *vs*. pSM214 at p < 0.01 (#). From 44 h on, difference was statistically significant *vs*. both controls at p < 0.01 (*). Comparison of survival percentiles showed a statistically significant difference (p < 0.01) between pSM539 and control groups pSM214 and PBS at 75% and 50% percentiles. Survival of control mice receiving pSM214 was never statistically different from that of mice receiving PBS (p > 0.2).

That IL-1Ra delivered by *B. subtilis *can have an anti-inflammatory protective effect *in vivo *was shown in a model of shock and death induced by bacterial endotoxin (LPS) in the mouse (Figure 5), an acute syndrome in which IL-1β plays a major role [14-16,25]. LPS-sensitive C3H/HeOuJ mice receiving recombinant pSM539 bacteria intra-peritoneally 24 hours before administration of a lethal dose of bacterial LPS could survive significantly longer than mice administered the control pSM214 bacteria or PBS, in agreement with previous data on the efficacy of IL-1Ra in inhibiting LPS-induced shock [13-15].

**Figure 6:**
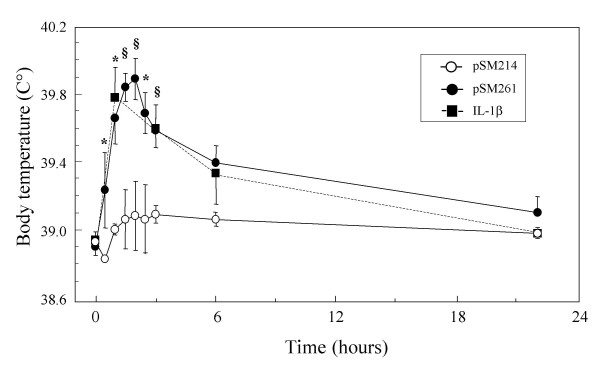
Intra-colonic administration of IL-1β-producing *B. subtilis *provokes fever and shock in rabbits. Increase in body temperature in rabbits receiving a single intra-colonic instillation of 1 × 10^9 ^live cells of *B. subtilis *pSM261 (producing IL-1β; ●) or pSM214 (β-lactamase control; ○). As positive control, rabbits received a single intra-colonic administration of 250 μg purified human recombinant IL-1β (■). Data are the mean ± SEM of values of 5 rabbits within a single representative experiment. In the group treated with IL-1β-producing pSM261 bacteria, 2/5 animals died within 30 h of hypotensive shock. From 0.5 to 3 h after treatment, body temperature of pSM216-treated rabbits was significantly higher than that of animals receiving control pSM214 (§p < 0.05;,* p < 0.01).

To validate the concept of delivery of bioactive recombinant proteins via the colonic mucosa by means of recombinant *B. subtilis*, another *B. subtilis *strain was constructed (pSM261, engineered for production of human mature IL-1β) and administered *in vivo *to rabbits. As shown in Figure 6, the intra-colonic administration of 1 × 10^9 ^live cells of *B. subtilis *pSM261 induced a significant increase in body temperature, superimposable to that caused by intra-colonic instillation of purified recombinant IL-1β. Furthermore, in agreement with the systemic effects of massive doses of IL-1β administered parenterally [25,28], intra-colonic administration of pSM261 caused shock and death in 9/14 animals (64%). It is concluded that the mucosal delivery of engineered *B. subtilis *in the large intestine is a suitable system for attaining significant blood levels of bioactive recombinant proteins and systemic effectiveness.

## Discussion

The use of live bacteria is very common in particular in vaccinology, where attenuated or mutant bacteria have been employed for decades as antigen carriers. The advantage of live bacteria relies on their capacity of colonising the host and enter the host organs/tissues with the same modalities as their virulent counterparts, thus eliciting the relevant immune response and immune memory, at variance with killed bacteria or purified bacterial components. Thus, attenuated strains of *Salmonella*, *Listeria monocytogenes*, *Mycobacterium tuberculosis*, *Vibrio cholerae *are being developed and used as vaccine carriers [29-31]. A further development in the use of live bacteria as antigen carriers in vaccination exploits the technologies of genetic engineering for introducing multiple antigens from different micro-organisms into a single non-virulent bacterial carrier (*e.g.*, food-grade lactic acid bacteria), with the possibility of including T- and B-stimulating epitopes from different antigens, and also to engineering into the same carrier adjuvant sequences derived for instance from an immunostimulating cytokine [30-34].

Among bacterial systems developed for antigen delivery in vaccination, some strains of non-pathogenic, food-grade or GRAS (generally regarded as safe) bacteria have been examined for the topical delivery of pharmacologically active protein drugs, after cell engineering with the DNA coding for the protein of interest. This is the case of *Lactococcus lactis *and of *Streptococcus gordonii*, which have been engineered to produce recombinant antibodies, adjuvant and anti-inflammatory cytokines, and used to deliver these proteins locally at the mucosal surface after oral administration [3-9]. The goal of these delivery approaches was that of making the recombinant proteins available for therapy of local pathologies or for local effects: antibodies for passive immunotherapy of local infections [3,4], cytokines as adjuvants for mucosal vaccines [5,6], inhibitory cytokines for anti-inflammatory therapy of localised chronic inflammatory diseases (IBD-like pathologies) [7-9]. Although undoubtely promising and susceptible of vast applications, the method of mucosal delivery of therapeutic protein through recombinant bacteria acting as cell factories needs further and deeper investigation. This should include the central issue of safety and contained/controlled release of recombinant micro-organisms [8], the problem of assessing the mucosal permanence of bacteria (extent and duration of colonisation depending on the changes in the mucosal environment in different conditons of health and nutrition) and the extent of protein release, and the issue of pharmacodynamics of the delivered protein in particular for its systemic effects, beyond the boundaries of the local delivery environment.

The delivery system proposed here is not based on the permanence/colonisation capacity of bacteria in the host mucosal surfaces, but it relies on the capacity of sporulating bacteria of releasing intracellular proteins in non-permissive environments. *B. subtilis *cells engineered to produce human IL-1Ra were able to release the recombinant protein (intact and biologically active) following a sporulation signal *in vitro *[24]. This observation could be repeated *in vivo*, when recombinant *B. subtilis *cells were inoculated in the intestine of rats or rabbits (a non-permissive environment that does not allow the vegetative life of *B. subtilis*). The recombinant protein could be detected locally shortly after administration of bacteria and persisted at measurable levels for several hours. Release and recovery of recombinant IL-1Ra was much more abundant and consistent in the large intestine as compared to the small intestine. Most interestingly, the recombinant protein released from sporulating bacteria delivered in the large intestine was absorbed in the bloodstream at detectable levels, whereas no circulating IL-1Ra could be found after bacterial delivery in the small intestine. IL-1Ra present in the blood was intact, as judged by its molecular mass in Western blotting, and retained full IL-1-inhibiting activity, as judged by its capacity of dose-dependent neutralisation of IL-1β *in vitro*. The passage of an intact protein from the intestinal lumen to the bloodstream is not a new concept. Indeed, transcytosis has been extensively described in intestinal epithelial cells, and allows transport of intact proteins and macromolecules from the intestinal lumen to the circulation through an endocytic non-degradative pathway in physiological conditions of integrity of the intestinal mucosal barrier [35-39]. This mechanism of transcytotic transport, quantitatively scarce as compared to the degradative pathway of protein absorption, may have a role in physio-pathological passage of antigens, allergens, and toxins.

Delivery of IL-1Ra through engineered sporulating bacteria apparently had some pharmacokinetics advantages as compared to the purified protein. Whereas the absorption into the bloodstream was quick after administration of the purified protein (T_max _at 60 min), IL-1Ra released from intra-colonically administered *B. subtilis *had a much slower kinetics of absorption (T_max _200 min), as expected by the fact that the protein must be released from bacteria before being absorbed. Furthermore, although the C_max _was decreased for *B. subtilis *IL-1Ra (136 ng/ml *vs. *482 ng/ml for the purified protein; only partially attributable to the higher dosage of the purified protein), the AUC/dose were almost identical. Thus, IL-1Ra delivered intra-colonically by *B. subtilis *is absorbed into the bloodstream at a slower and more constant rate than the purified protein delivered in the same site, which is absorbed quickly into the bloodstream and rapidly disappears thereafter. Thus, it appears that bacteria do not undergo sporulation all at the same time (which would result in a rapidly appearing and disappearing peak of protein), but release the protein constantly from the moment of administration for about 8 h. This would allow a controlled and sustained circulating level of the protein, thus a more favourable pharmacodynamic profile, with a single administration.

The protein selected for *in vivo *delivery with *B. subtilis *is the IL-1 receptor antagonist IL-1Ra, a competitive non-activating ligand of the IL-1 receptor with IL-1 inhibitory activity [11,12]. IL-1 is a potent inflammatory cytokine which, in pathological conditions, is responsible of chronicisation of inflammation, tissue destruction, organ failure, hypotensive shock [10]. Anti-IL-1 strategies have been attempted in acute an chronic inflammatory diseases with the use of recombinant IL-1Ra protein [11,12]. The poor outcome of clinical trials in septic shock has highlighted the problems of a therapy based on the systemic administration of a purified recombinant protein, whose efficacy is hampered by its rapid pharmacokinetics [11,12,18]. At present, experimentation of therapeutic IL-1Ra is being targeted to slowly progressive chronic diseases with defined organ/tissue targets (*e.g.*, rheumatoid arthritis) [40,41]. To achieve sustained IL-1Ra levels, gene therapy approaches have been attempted with promising results in animal models of experimental arthritis, ischemic brain damage, autoimmune diabetes [19-23]. However, the risk remains of side effects due to the uncontrolled inhibition of the physiologically important IL-1 activity. Indeed, a precise balance between between IL-1β and IL-1Ra should be maintained for achieving proper tissue homeostasis, as shown for the intestinal mucosa [42].

The drug delivery strategy here described merges the well-known approach of vaccination with live bacteria with that of gene therapy. The delivery of pharmacologically active proteins by live sporulating bacteria, as described here, presents a series of advantages over other similar approaches. At variance with conventional gene therapy, the gene coding for the drug protein is introduced in a bacterial carrier rather than in host cells, a situation that would allow a complete control of its permanence in the body. In a previous study, intragastric or vaginal administration of *Streptococcus gordonii *engineered to release human IL-1Ra resulted in a prolonged local delivery of the protein, consequent to the capacity of *S. gordonii *to colonise the mucosal surfaces [9]. Mucosal delivery of IL-1Ra (by intragastric administration of engineered *S. gordonii*) also had a local therapeutic effect in a model of ulcerative colitis [9]. The delivery system with sporulating bacteria described here differs from that with *S. gordonii*, as it causes rapid local release of the recombinant protein (*e.g. *in the large intestine, where IL-1Ra peaks at 4 h and decreases towards background at 24 h), followed by absorption into the bloodstream. In preliminary experiments in the mouse, IL-1Ra-expressing bacteria were also administered intragastrically or subcutaneously. This achieved appearance of human IL-1Ra in the serum, and systemic effects of inhibition of LPS-induced shock and death (data not shown). This is a new finding, that opens the possibility of exploiting localised bacterial administration (*e.g. *at mucosal sites) for systemic drug delivery. The amount of protein released at the mucosal site directly correlates with the number of administered bacteria, since the internal body environment does not sustain bacterial replication but induces sporulation. This allows an exact control of the dose of drug delivered and, based on the pharmacokinetics parameters, of the blood levels that can be reached. The same result could not be easily obtained with *S. gordonii*, as amount and timing of protein release may be influenced by variation of the colonisation capacity depending on variations of environmental conditions of the host tissues.

A problem that should be faced when using recombinant bacteria *in vivo *for therapy or vaccination is that of safety and contained release of genetically modified organisms (GMO). The use of suicidal genes or the deletion of genes vital for survival outside the host organism have been explored with very promising results [8,43]. The bacterial system proposed here can be modified in the sporulation mechanism for the control of its survival. In preliminary experiments, the recombinant *B. subtilis *pSM539 strain was engineered in order to inactivate a gene involved in sporulation control. As a consequence, in response to *in vitro *sporulation signals (adverse environmental conditions) the mutated Spo^- ^strain could regularly initiate the sporulation process, undergoing cell autolysis and release of the cytoplasmic proteins (including the recombinant IL-1Ra), but it was incapable of eventual spore formation and further survival. Likewise, release of the recombinant protein from Spo^- ^*in vivo *was comparable to that of Spo^+ ^bacteria, but spores could never be recovered from intestinal lavage and faeces (data not shown). This suggests that the system can be optimised to full biological containment and environmental safety without altering its delivery properties.

## Conclusions

The novel system of protein drug delivery here proposed links some of the advantages of gene therapy (endogenous production of the relevant protein, targeted delivery) to the possibility of controlled release in terms of timing and protein amount. Exploitation of the mechanism of bacterial autolysis in non-permissive environments allows release of intracellular proteins, including the known amount of the pharmacologically active recombinant protein drug. The release is persistent for several hours, allowing to maintain more constant protein levels in the bloodstream. The system is simple, cheap, and can be developed to full environmental safety (*i.e.*, avoiding the risk of release of genetically modified bacteria in the environment).

The concept that pharmacologically active proteins released at the colonic mucosal surface can be absorbed and reach the circulation intact and retaining full activity (validated with two proteins with opposite effects, IL-1Ra and IL-1β) opens promising avenues to the use of local delivery for the therapy of systemic diseases.

## Methods

### Bacterial strains

Engineered *B. subtilis *strains were constructed as previously described in detail [44,45]. Briefly, cDNA coding for mature human IL-1Ra (encompassing the mutation N91>R), and cDNA coding for mature human IL-1β were cloned between *Eco*RI and *Hind*III in pSM214, a *B. subtilis *plasmid which promotes the synthesis of recombinant products intracellularly, to obtain recombinant plasmids pSM539 (carrying the cDNA for IL-1Ra) and pSM261 (carrying the cDNA for IL-1β). Plasmids were used to transform the *B. subtilis *SMS118 strain. The pSM539-harbouring *B. subtilis *strain SMS118(pSM539) could produce 1.0–2.0 mg IL-1Ra/10^9 ^cells/0.35–0.49 g (wet weight), after conventional culture overnight in 1 liter flasks. The SMS118(pSM261) strain in the same culture conditions produced 0.15–0.25 mg IL-1β/10^9 ^cells/0.35–0.49 g. As negative control, *B. subtilis *strain SMS118 was transformed with the pSM214 plasmid, which contains the gene of β-lactamase (conferring resistance to penicillin). All strains were leu^-^, pyrDI, npr^-^, apr^-^. Sporulation-defective (Spo^-^) strains were constructed by mutation in the *srfA *gene, as previously described [46] and were kindly provided by Dr. G. Grandi (Chiron S.r.l., Siena, Italy).

### Bacterial preparations

Bacteria were grown in LB medium containing 5 mg/l chloramphenicol for 7 h at 37°C under shaking and harvested by centrifugation (3,000 × g, 20 min, 4°C). For sporulation supernatant preparation, 0.25 g wet weight of bacteria (corresponding to 0.5–0.7 × 10^9 ^cells) were suspended in Difco sporulating medium (bacto beef extract 3 g/l, peptone 5 g/l, NaOH 0.25 mM, MgSO_4 _10 mM, KCl 0.1%, MnCl_2 _0.1 mM, Ca(NO_3_)_2 _1 mM, FeSO_4 _1 mM, pH 6.8) without chloramphenicol and incubated at 35°C with shaking. Aliquots of sporulation supernatant were harvested by centrifugation (14,000 × g, 5 min) at different time points. Following sporulation signals, both Spo^+ ^and Spo^- ^bacteria initiate the autolysis process, which ends in cell autolysis with release of cytoplasmic content. However, whereas in Spo^+ ^bacteria there is formation of a spore with preservation of strain survival, Spo^- ^bacteria are unable to form a spore thus undergoing complete cell destruction (Figure 1A). Upon sporulation signals, both Spo^+ ^and Spo^- ^bacteria released 100% of intracellular recombinant products in a time-dependent fashion, with maximal relaease between 2 an 8 h (Figure 1C) [24].

### SDS-PAGE analysis

Protein samples were run on 13.5% mini SDS-PAGE according to Lämmli [47] and stained with Coomassie R-250. The gel was subjected to laser scanning on a Molecular Dynamics Personal Densitometer, and the densitometric analysis was made using Image Quant software (Molecular Dynamics, Sunnyvale, CA).

### Animals

Experimental animals were: female C3H/HeOuJ mice of 10–12 weeks of age (20–25 g) (for all *in vivo *experiments), female C3H/HeJ mice of 5–8 weeks of age (for thymocyte proliferation), female Sprague-Dawley rats (around 300 g), and female New Zealand rabbits (1.9–2.5 kg). All animals were purchased from Charles River Italia (Calco, Italy) and were housed in standard cages at 22 ± 1°C with 12 h light-12 h dark cycle. Animals received standard diet and tap water *ad libitum*.

### *In vivo *administration of *B. subtilis*

Bacteria were harvested and resuspended in LB medium or sterile PBS.

Mice received a single intra-peritoneal injection of 0.2 ml of bacterial suspension in PBS.

Rats were fasted overnight before the surgical procedure and maintained under urethane anaesthesia throughout. Bacteria (in LB medium diluted 1:1 in PBS) were instilled in the small intestine (duodenum) with a 22 1/2 G needle, in a volume of 1–10 ml. Two surgical ligatures were applied, one at the beginning of the duodenum immediately below the needle entry puncture (to avoid exit of instilled bacteria), and another one near the ileocecal valve, to limit to the small intestine the transit of bacteria. Intra-colon instillation was performed again with a 22 1/2 G needle in the ceacum immediately below the ileo-caecal valve, in a volume of 5–10 ml. Two surgical ligature were applied just below the needle entry point and at the colon terminal region, to avoid loss of bacteria. Animals were sacrificed by exanguination at different times after treatment, to collect blood and intestinal washings.

For intra-colonic administration of bacteria in rabbits, animals were fasted overnight prior to treatment, then lightly restrained in conventional stocks and maintained conscious throughout the experiment. A rounded-tip urethral catheter (Rüsh, Germany) was carefully inserted 10 cm into the distal colon via the anal route and 2 ml of *B. subtilis *suspension were administered. Serum samples were prepared from blood collected from the rabbit marginal ear vein at different times (0–8 h) after intra-colonic administration of bacteria. In some experiments, animals were sacrificed, to collect the large intestine content (saline washing).

Protocols of animal experimentation were reviewed by the institutional ethical board for adherence to ethical guidelines for animal research conduct (Italian D. L.vo 27/01/1992 n. 116 and corresponding EU directive 86/609; policy of refinement, reduction and replacement towards the use of animals for scientific procedures 99/167/EC – Council Decision of 25/1/99), and previously authorised by the Italian Ministry of Health.

### Detection of human IL-1Ra in animal samples

Western blotting: samples were subjected to reducing 15% mini SDS-PAGE and analysed by Western blotting using a polyclonal rabbit serum anti-human IL-1Ra and a goat anti-rabbit IgG secondary antibody conjugated with horseradish peroxidase, as described in detail elsewhere [48]. Serum samples were filtered on Microcon 100 (MWCO 100,000; Amicon, Beverly, MA) before analysis.

ELISA measurement: samples were subjected to quantitative determination of human IL-1Ra using a specific ELISA (Amersham, Little Chalfont, UK), following the manufacturer's instructions. The lower detection limit was 20 pg/ml. Purified human recombinant IL-1Ra was used as standard. Serum samples were filtered on Microcon 100 (Amicon) before analysis.

Biosensor measurement: detection of IL-1Ra in serum samples and intestinal washings was confirmed with the biosensor BIAcore™ system (Pharmacia Biosensor AB, Uppsala, Sweden), which allows real time biospecific interaction analysis by means of the optical phenomenon of surface plasmon resonance, as previously described in detail [49]. The lower detection limit for human IL-1Ra was 2 pg/ml.

### IL-1-induced thymocyte proliferation

The classical assay of co-stimulation of murine thymocyte proliferation was used to evaluate the bioactivity of IL-1 and IL-1Ra. Briefly, thymocytes from 5–8 week-old C3H/HeJ mice (preferentially used because of their LPS unresponsiveness) were cultured at 6 × 10^5 ^cells/well of Cluster^96 ^plates (Costar, Cambridge, MA) in 0.2 ml of RPMI-1640 medium (Life Technologies, Paisley, Scotland) supplemented with 2 mM L-glutamine, 25 mM HEPES buffer, 50 μg/ml gentamycin sulfate, 1.25 × 10^-5 ^M 2-ME (all from Sigma Chemical Co.), 5% fetal bovine serum (Hyclone, Logan, UT) for 72 h in moist air with 5% CO_2 _[50]. The biological activity of IL-1β was assessed as co-stimulation of thymocyte proliferation, by adding to the culture wells a selected amount of human recombinant IL-1β (30–300 pg/ml) [51] and a suboptimal concentration of purified PHA (1.5 μg/ml; Murex Diagnostics, Dartford, UK). Cells were then pulsed for 18 h with 18.5 kBq/well of [^3^H]TdR (sp. act. 185 GBq/mmol; Amersham) and their proliferation was measured as radiolabel incorporation with a β-counter.

The biological activity of IL-1Ra was evaluated as inhibition of IL-1β-dependent thymocyte proliferation. To this end, cells were stimulated to proliferate (with IL-1β and PHA) in the presence of increasing concentrations of human recombinant IL-1Ra [51] or serial dilutions of serum from rabbits receiving pSM214 or pSM539 intra-colonically, or from untreated rabbits. The concentration of IL-1Ra in serum of pSM539-treated rabbits was determined by ELISA and serum was added to the cultures after appropriate dilution. Control sera from pSM214-treated or untreated rabbits were used at the same dilutions as IL-1Ra-containing serum. Cell proliferation was then evaluated as radiolabel incorporation as described above.

To assess that the effect of IL-1Ra-containing serum was indeed due to IL-1Ra, a polyclonal rabbit antibody against human IL-1Ra [48] was added to the cultures at a dilution of 1:300, *i.e. *the dilution previously found to inhibit 50% of the activity of 10 ng/ml IL-1Ra in the thymocyte assay (not shown).

### IL-1-induced fever

Rabbits were lightly restrained in conventional stocks throughout the experiment, and accustomed to the stocks over a period of 2 h, to minimise variations in body temperature. Body temperature was measured by means of a cutaneous thermistor probe (TM-54/S and TMN/S; LSI-Lastem, Settala Premenugo, Italy) placed between the left posterior paw and the abdomen and allowed to stabilise for 2 min. *B. subtilis *suspensions (2 × 10^9 ^live cells/rabbit) were instilled in the distal colon 1 h before i.v. administration of 50–100 ng/ml highly purified LPS-free human recombinant IL-1β in pyrogen-free saline through the marginal ear vein. Temperature was recorded every 20 min for 3 h starting from IL-1β administration. In experiments with IL-1β-producing strain pSM261, rabbits received an intra-colonic administration of 1 × 10^9 ^pSM214 (control) or pSM261 (IL-1β) bacteria, or 250 μg purified human IL-1β. Temperature was recorded up to 22 h after treatment.

### IL-1-induced neutrophilia, hypoferremia, hypoglycemia

Live cells of *B. subtilis *strains pSM214 and pSM539 were instilled in the distal colon (1 × 10^9 ^cells/kg), 2 h before administration of IL-1β. Blood samples were drawn 2, 4, 6, 8 and 24 h after intra-peritoneal inoculum of 0.1 μg/kg human recombinant IL-1β. The number of circulating neutrophils was assessed by flow cytometry. The plasma iron concentration was determined colorimetrically with a commercially available kit (Fe; Boehringer Mannheim, Mannheim, Germany). Hypoferremia (60–75% decrease of plasma iron level) was evident from 4 to 24 h after IL-1β inoculum. The blood glucose concentration was measured in serum samples by the glucose/glucose oxidase/peroxidase method with commercially available kits (Glucose GOD Perid; Boehringer Mannheim) or by biosensor detection with devices for diagnostic monitoring (Roche Diagnostics, Milano, Italy). Overlapping results were obtained in rats and rabbits.

### LPS-induced shock in the mouse

LPS-sensitive C3H/HeOuJ mice received an intra-peritoneal inoculum of 0.5 ml PBS alone or containing bacterial suspensions (control pSM214, IL-1Ra-producing pSM539; 3 × 10^6 ^bacteria/mouse), 24 h before i.p. administration of 15–20 mg/kg of LPS (from *E. coli *055:B5; Sigma Chemical Co., St. Louis, MO). LPS inoculum was delayed to 24 h after bacteria administration to avoid interference of pre-inoculum. In fact, preliminary experiments showed that intra-peritoneal inoculum of PBS decreased significantly LPS toxicity when administered at shorter times before LPS (data not shown). Mice were observed for 7 days after LPS administration and deaths recorded.

### Statistical analysis

Results are presented as mean ± SEM. Statistical significance was assesed by two-tailed Student's *t *test. Comparison of survival curves was performed by the χ^2 ^test. Calculation of percentiles was performed by survival analysis. All calculations were performed with the Stratgraphics Plus 5 programme (Manugistics, Inc., Rockville, MD).

## List of abbreviations

IL, interleukin; IL-1, interleukin-1; IL-1Ra, interleukin-1 receptor antagonist; IBD, inflammatory bowel disease; LPS, bacterial lipopolysaccharide, AUC, area under the curve; PMN, polymorphonuclear leukocytes; GRAS, generally regarded as safe; GMO, genetically modified organisms.

## Authors' contributions

SP carried out the *in vivo *and pharmacokinetics studies in rats and rabbits, and performed the statistical analysis.

PB designed and performed the bioactivity studies.

PR designed and performed the microbiological and biochemical work.

DB coordinated the bioactivity studies, organised the data, and wrote the manuscript.

AT designed and coordinated the entire study.

All authors read and approved the final manuscript.

## Figures and Tables

**Table 1:** Local and systemic IL-1Ra after administration of IL-1Ra-producing *B. subtilis*

	**Delivery of *B. subtilis *pSM539**		**Detection of IL-1Ra**	
	
**Animal**	**Route**	**Time**	**Local**	**Serum**
Mouse	peritoneal cavity	0 h	-	-
		3 h	++	+
		6 h	+	-
		24 h	-	-
Rat	small intestine	0 h	-	-
		3 h	++	-
		4 h	+ (B: 149.4 μg)	-
		6 h	+/-	-
		8 h	+/- (B: 16.5 μg)	-
		24 h	-	n.t.
	large intestine	0 h	-	-
		2 h	n.t.	++ (B: 0.6–1.3 μg/ml)
		3 h	+++	n.t.
		4 h	+++	++ (B: 0.5–1.9 μg/ml)
		6 h	++	+
		8 h	++	+ (B: 0.1–0.4 μg/ml)
		24 h	+	n.t.
Rabbit	large intestine	0 h	-	- (B/E: 0 μg/ml)
		0.5 h	n.t.	+ (B: 0.2–0.4 μg/ml)
		1 h	n.t.	++ (B/E: 0.2–1.2 μg/ml)
		2 h	n.t.	++ (B/E: 0.6–1.6 μg/ml)
		3 h	n.t.	++ (B: 0.6–2.0 μg/ml)
		4 h	++	++ (B/E: 0.4–2.1 μg/ml)
		6 h	n.t.	++ (B/E: 0.3–0.9 μg/ml)
		8 h	++	++ (E: 0.6–2.4 μg/ml)
		24 h	n.t.	+ (B: 0.3 μg/ml)

Mice, rats and rabbits were administered live cells of *B. subtilis *strain pSM539 (engineered to produce human mature IL-1Ra; 1 × 10^8^-2 × 10^9 ^cells/kg) into the peritoneal cavity, the small intestine, or the large intestine. Animals were sacrificed at different time points after bacterial inoculum, and samples of serum and intestinal washings were taken. The presence of human IL-1Ra was assessed in all samples by Western blotting. Semi-quantitative analysis was performed after laser scanning densitometry in comparison to different amounts of standard human recombinant IL-1Ra, and scored as follows: -, no detection; +/- very faint and/or inconsistent detection; + consistently positive; ++ abundant detection; +++ very high levels. Western blotting analysis revealed that IL-1Ra recovered from serum and intestinal washings had the same molecular mass as the standard IL-1Ra. No proteolytic fragments or larger aggregates could be detected. For some samples, quantitative assessment of IL-1Ra was performed by ELISA (E) or by BIAcore (B) analysis. Assessed animals were 2–16/group/time. n.t., not tested.

**Table 2:** Pharmacokinetic analysis of IL-1Ra in rabbit serum after administration of IL-1Ra-producing *B. subtilis*

**Intra-colon instillation**	**IL-1Ra (mg/kg)**	**C_max _(μg/ml)**	**T_max _(min)**	**AUC (0–6 h)/dose (g × h)/ml**
IL-1Ra	1.00	0.482	60	1.34
IL-1Ra + pSM241	1.00	0.372	60	1.05
pSM539	0.44	0.136	200	1.10

Groups of four rabbits received an intra-colonic instillation of highly purified human recombinant IL-1Ra (1.0 mg/kg) alone or admixed with control pSM214 bacteria (4 × 10^8 ^cells/kg), or of IL-1Ra-producing pSM539 bacteria (4 × 10^8 ^cells/kg). Serum samples were taken just before treatment (time zero) and after 1, 2, 4, and 6 h from intra-colonic instillation. The presence of human IL-1Ra in serum samples was determined by ELISA.
